# 2D Nanocomposite Membranes: Water Purification and Fouling Mitigation

**DOI:** 10.3390/membranes10100295

**Published:** 2020-10-20

**Authors:** Lara Loske, Keizo Nakagawa, Tomohisa Yoshioka, Hideto Matsuyama

**Affiliations:** 1 Department of Environmental, Process & Energy Engineering, Management Center Innsbruck (MCI)—The Entrepreneurial School, Maximilianstrasse 2, 6020 Innsbruck, Austria; ll9769@mci4me.at; 2 Research Center for Membrane and Film Technology, Department of Chemical Science and Engineering, Kobe University, 1-1 Rokkodai, Nada, Kobe 657-8501, Japan; 3 Research Center for Membrane and Film Technology, Graduate School of Science, Technology and Innovation, Kobe University, 1-1 Rokkodai, Nada, Kobe 657-8501, Japan; tom@opal.kobe-u.ac.jp

**Keywords:** nanosheet, water purification, antifouling, stacked membrane, mixed matrix membrane, photocatalysis

## Abstract

In this study, the characteristics of different types of nanosheet membranes were reviewed in order to determine which possessed the optimum propensity for antifouling during water purification. Despite the tremendous amount of attention that nanosheets have received in recent years, their use to render membranes that are resistant to fouling has seldom been investigated. This work is the first to summarize the abilities of nanosheet membranes to alleviate the effect of organic and inorganic foulants during water treatment. In contrast to other publications, single nanosheets, or in combination with other nanomaterials, were considered to be nanostructures. Herein, a broad range of materials beyond graphene-based nanomaterials is discussed. The types of nanohybrid membranes considered in the present work include conventional mixed matrix membranes, stacked membranes, and thin-film nanocomposite membranes. These membranes combine the benefits of both inorganic and organic materials, and their respective drawbacks are addressed herein. The antifouling strategies of nanohybrid membranes were divided into passive and active categories. Nanosheets were employed in order to induce fouling resistance via increased hydrophilicity and photocatalysis. The antifouling properties that are displayed by two-dimensional (2D) nanocomposite membranes also are examined.

## 1. Introduction

Safe drinking water is a basic human need, but it is only available to 71% of the worldwide population. In other words, 844 million people do not have basic water services [1]. Additionally, approximately four-billion people are affected by severe water scarcity for at least one month every year. Taking climate change, as well as the projected population growth, into consideration, an increasing number of people will be affected by sources of unsafe drinking water. The strain on water resources is perpetuated by the estimated annual increase in global water consumption of about 1%. In order to address the pressing issue of the demand for safe drinking water, goal number 6 of the Sustainable Development Goals (SDGs) that were developed by the United Nations is to ensure access to safe water and sanitation to all by 2030 [2].

Another rising problem is the increasing contamination of freshwater resources that people depend on for drinking water. Pollutants like organic waste, pathogens, fertilizers and pesticides, heavy metals, and Contaminants of Emerging Concern (CEC) are present in waters all around the world [3,4,5]. The treatment technologies that are intended to purify water must be based on the required quality of treated water and on the pollutants already present in existing water sources. For example, surface water has high microbial concentrations with compositions that vary greatly depending on upstream activities [6]. To purify water sources, the implementation of conventional water treatment solutions are comprised of coagulation, followed by flocculation, sedimentation, and disinfection as the final step [7]. However, the downsides of these technologies include their size, use of chemicals, and limited removal capacity. New technologies have begun to shift into the center of attention of researchers. Although alternative solutions, like advanced oxidation processes (AOP), yield high removal rates, they are still confined to a laboratory scale and produce undesired disinfection-by-products (DBP) [8]. In contrast, membranes are now being broadly implemented and they are beginning to be considered a viable alternative due to factors, such as low energy demand, small footprint, and simple operation [9].

Additionally, membranes can effectively remove CEC from water bodies [10]. However, drawbacks such as permeability-selectivity trade-off, stability, and fouling have limited the development and implementation of membranes [9]. Fouling refers to a deposition on membrane surfaces that are prone to adsorption, pore blocking, cake layer formation, and concentration polarization of contaminants. These effects result in a pressure increase and a simultaneous decline in pollutant rejection, flux, and membrane lifetime [11]. Fouling is caused by several water constituents that are categorized as organic-, inorganic-, or bio foulants. Because fouling is an interplay of several deposition mechanisms and materials, it is a complicated and case specific phenomenon. In order to implement the appropriate measures or membrane modifications, a sound understanding of the underlying fouling mechanisms is required [12]. Several methods have become common strategies to reduce the effect of fouling: chemical [13], physical [14], biological [15], and electrical [16]. Chemical methods comprise the application of cleaning agents, such as bases (NaOH, NaClO), acids (HCl, H_3_PO_4_, C_6_H_8_O_7_), surfactants (SDS), and disinfectants (H_2_O_2_, HNO_3_) to remove adsorbed and pore blocking foulants [17]. Physical methods describe the removal of weakly adhered foulants by back-flushing, by improving module designs, and by temporarily altering the processing conditions [17,18]. Although these approaches alleviate fouling, they require large areas for feed pre-treatment, and result in an increase in operation cost or complexity. Chemical cleaning reduces the membrane lifetime due to chemical or structural damage [19,20,21]. Therefore, developing membranes with intrinsic antifouling properties is crucial.

In recent years, nanomaterials have received considerable attention for superior properties, such as antimicrobial activity, conductivity, photocatalytic effect, and light-induced hydrophilicity in the case of TiO_2_ [22]. Nanocomposite membranes are known to increase stability, permeability, rejection, and alleviate fouling. The coating of surfaces or incorporation of nanoparticles is well documented in the literature [23]. Drawbacks for the use of nanoparticles, such as TiO_2_ in membranes, include pore-blocking, the agglomeration of the nanoparticles, degradation of the membrane via a photocatalytic defect, and leaching of the nanoparticles. By contrast, two-dimensional (2D) nanomaterials have shown promise as building blocks or fillers for membranes, due to their mechanical strength, flexible structure, chemical inertness, and separation performance [24]. Such 2D nanomaterials are so-called nanosheets and they are characterized by their high surface areas and an atomic thickness that exposes all of the ions to the surface, thus enabling electronic coupling [25]. Their fabrication methods, performances, and separation mechanisms as membrane materials have been thoroughly surveyed [26,27]. In general, the behavior of nanomaterials differs from that of bulk materials and depends on the particular nanostructure [28]. Due to their large surface area, nanosheets improve both the surface roughness and hydrophilicity in addressing organic fouling [29]. Additionally, the presence of nanosheets in TFC membranes has demonstrated improvements in chlorine resistance [30,31]. However, the applications of 2D nanomaterials for antifouling properties have only been limited to graphene-based nanosheets in previous reports [32,33].

**Figure 1:**
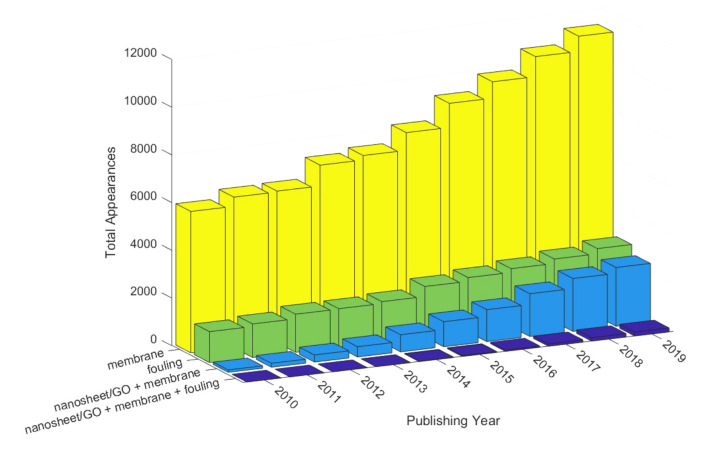
Increasing interest in antifouling two-dimensional (2D) nanocomposite membranes; results were obtained from the “Web of Science”.

In the present work, a broad range of antifouling nanosheet composite membranes were analyzed in order to better understand the role of 2D nanostructured materials for water purification applications. An analysis of the appearance of keywords related to nanosheets in “Web of Science” demonstrated increasing interest in membranes, nanosheet membranes, fouling, and nanosheet membrane fouling. This upward trend could be observed over the past 10 years, as demonstrated in Figure 1. Research that is focused on antifouling nanosheet membranes is still rare; however, the interest in the field of nanosheet/GO membranes grows annually and will continue to do so. In this paper, we review the utility of 2D nanocomposite membranes with antifouling properties for water purification. Nanosheet-based mixed matrix membranes and stacked membranes with antifouling properties for oil, organic, and inorganic compounds are compared. Based on the growing interest in 2D nanomaterials for fouling mitigation, this review should give researchers a summary of the current achievements in the field. Furthermore, the present work should suggest where to direct future work.

## 2. 2D Nanosheet Membranes

### 2.1. Nanostructured Materials in Membrane Technology

In recent years, the properties of nanostructured materials have been increasingly investigated for their use in fuel cells, catalysis, energy storage, coating applications, and membrane separation. Pokropivny and Skorokhod [34] classified nanostructures into 37 groups that were defined by the following general dimensions: 0D, nanoparticles, or nanodots; one-dimensional (1D) nanowires, nanostrands, or nanotubes; and, 2D nanosheets or nanoplates. At least one of the three dimensions is smaller than 100 nm in order to be considered a nanostructure [35].

The blending of inorganic nanofillers with polymers allows hybrid membranes to combine the cost-effectiveness and superior permselectivity of polymers and the outstanding mechanical, thermal, and chemical stability of ceramics [36]. However, high concentrations of low specific surface nanoparticles tend to form aggregates that reduce the advantageous effects of nanomaterial modification. Another drawback with the use of nanoparticles is their loss during membrane preparation [37]. One-dimensional carbon nanotubes (CNT) are an alternative to nanoparticles, and expected to demonstrate ultrafast water flux in nanochannels, according to simulations [38]. Nevertheless, the performance of carbon nanotubes has been limited to theoretical studies [36]. In contrast to other nanomaterials, nanosheets stand out, due to their chemical inertness, flexibility, and physio-chemical and optoelectronic properties. In the field of membrane technology, the application of nanosheets yields high separation, mechanical strength, and it forms flexible structures [24].

### 2.2. Nanosheet Materials

Graphene-based nanomaterials (GBN) are the most popular group of nanosheets and they are subject to ongoing, intensive studies [39]. Based on the promising properties of GO nanostructures, new nanosheets are continuously being discovered, and the variety of their molecular structure has extended the possibilities for applications. Despite promising properties, GO has weak Van der Waals attractive forces, which render them unstable in water. Other materials that have received attention in the field of membrane science include Covalent Organic Frameworks (COF), Metal Organic Frameworks (MOF), Transition Metal Dichalcogenides (TMD), Layered Double Hydroxide (LDH), Boron Nitride (BN), and graphitic carbon nitride [40,41,42,43]. In this review, only nanosheet materials that have been tested for antifouling propensity will be mentioned.

**Figure 2:**
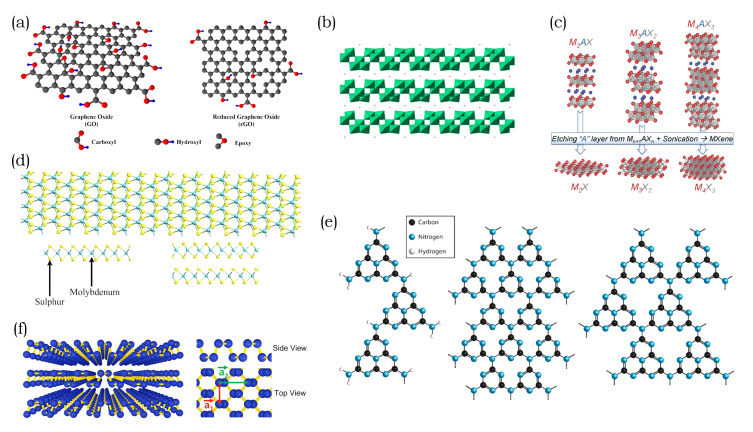
Molecular structures of different 2D nanomaterials. (**a**) GO and rGO nanosheets. Reproduced with permission from [47], published by MDPI AG. License CC-BY. (**b**) Layered structure of HNb_3_O_8_ with alternating sheets of interconnected NbO_6_ and protons. Reprinted with permission from [48]. Copyright 2017 American Chemical Society. (**c**) The 3 major MAX Phases and their respective MXene nanosheets. Copyright (2013) Wiley. Used with permission from [49]. (**d**) MoS_2_ nanosheet top view and side view of nanosheet single as well as double layers. Adapted with permission from [50], published by Springer Nature. License CC-BY. (**e**) From left to right, Liebig’s melon, fully condensed triazine C_3_N_4_, and predicted structure, which is fully condensed polyheptazine (tri-s-triazine) C_3_N_4_. Adapted from [51] with permission of the PCCP Owner Societies. License CC-BY. (**f**) Cross-sectional view from phosphorene nanosheets on the left and top, and side views on the right bottom. Adapted with permission from [52]. Copyright (2014) American Chemical Society.

GBNs are the most prevalent group of nanomaterials concerning water purification, as previously mentioned. Graphene is the parent material of all other derivatives including fullerene, CNTs, GO, and rGO. In its 2D nanosheet structure it is a conductor, similar to metals, with zero band gap. Graphene oxide is an intensively studied material with promising attributes for several different applications and a low-cost alternative to pristine graphene [44]. The structure of graphene oxide resembles a honeycomb lattice with functional groups on the edges or around holes. Oxidized $sp^{3}$ and pristine graphitic $sp^{2}$ regions are randomly distributed in the basal plane of the nanosheet, as depicted in Figure 2a. GO nanosheets have defects that originate from their synthesis or subsequent modifications. The dominant functionalities are hydroxyl and epoxy groups, whereas carbonyls are located at the edges and in holes [45]. It is favorable to synthesize GO nanosheets that consist entirely of $sp^{2}$ regions. In removing the oxygen-containing groups of the $sp^{3}$ regions, the chemical, electrical, and optical properties of GO nanosheets are altered in order to improve the fit for their intended use [46].

Although less prominent than GBNs, 2D transition metal oxides have become an important material for membrane modifications. Especially, TiO_2_ has found widespread application due to its intrinsic properties and cost effectiveness [53]. The group of transition metal oxide nanosheets is diverse, with many of these materials exhibiting a variety of properties. In terms of catalytic performance, popular nanosheets are BiVO_4_, MnO_2_, TiO_2_, Fe_3_O_4_, NiO, and WO_3_ [54]. In particular, SnO_2_, ZnO, WO_3_, V_2_O_5_, Nb_2_O_5_, Nb_2_O_5_ doped TiO_2_, SrTiO_3_, and NaNbO_3_ demonstrate an oxidative effect and/ or superhydrophilic wettability, which is induced by their photocatalytic properties [22]. In contrast, transition-metal layered oxides, such as titanium and niobium-based metal oxide (Figure 2b), exhibit electrical conductivity, photocatalytic activity, and strong acidity [55].

Similar to TMOs, new MXene nanosheet materials are continuously discovered and they contribute to the growth of these rather diverse groups of 2D nanomaterials. The group of MXene consists of recently discovered 2D nanomaterials. Their hydrophilic surface and laminar structures make them suitable for membrane applications. Nanosheets that belong to this group are exfoliated from the so-called MAX phase. The bulk material of MXene consists of layered ternary carbides as well as nitrides and it is described by the formula M_n+1_AX_n_. In general, n is equal to 1, 2, or 3; M is an early transition metal; A is an element mostly from group 13 or 14; and, X represents either C and/or N. The A-elements are interlayers of the M-element, and X-elements fill its octahedral sites. Figure 2c describes the structures of 3 MAX phases and their respective nanosheets after etching. The most investigated material in the MAX phase is Ti_3_AlC_2_ with M and A representing Ti_3_C_2_ and Al, respectively [56]. The nanosheet that was obtained when exfoliating this MAX phase is Ti_3_C_2_T_*x*_. T represents the terminating groups, and with O, OH, and/or F, and x indicating the number of terminating groups [57]. The different terminating groups and their composition directly impact the properties of nanosheets. When considering the attention that MXene materials have received in such a short time after their discovery, it is expected that there will be more breakthroughs in the future [49,58].

Transition Metal Dichalcogenides was the first group of nanosheets that sparked research output, after the discovery of graphene. Until today, it is one of the most investigated nanosheet materials for nanocomposite membranes [59]. TMDs have a laminar structure that can undergo exfoliation to form 2D nanostructures with the generalized formula MX_2_. Here, M and X represent a transition metal of the groups 4 to 10 and a chalcogen, respectively. Metal atoms form a hexagonal layer that is embedded between two layers of chalcogenide atoms, as illustrated in Figure 2d. A large number of possible chemical structures render this group of nanosheets highly versatile, with utility ranging from catalysis to energy storage to membrane separation [60]. In general, TMDs have many polymorphs with 1T, 2H, and 3R being the most commonly encountered structures. Here, the digits represent the number of MoS_2_ layers and the letters represent the trigonal, hexagonal, and rhombohedral structures, respectively [61].

Unlike TMDS, carbon nitride nanosheets are a comparably young group of materials with highly promising photocatalytic properties [62,63]. Their discovery resulted in an immediate surge in carbon-nitride related research activity. These nanosheets are usually referred to as graphitic carbon nitride or g−C_3_N_4_ with s-heptazine and s-triazine units. This material terminology is considered misleading by Miller et al. [51]. In their review, they state that most nanosheets described in the literature are actually polymeric C_x_N_y_H_z_ structures. These are similar to Liebig’s melon, but consist of ribbon-like heptazine units and are linked via NH or $sp^{2}$-bonded N atoms, as described in Figure 2e. According to their research, true g−C_3_N_4_ nanomaterials have only been reported by Algara-Siller et al. [64] and Kouvetakis et al. [65]. Miller et al. [51] suggested use of the term gCN(H) for nanostructures obtained via the routes of thermolysis or reaction of precursors in order to avoid misleading nomenclature. This naming indicates a large amount of H present and more or less condensation of the polymeric structure.

An alternative to semiconducting graphitic carbon nitrides involves the recently discovered laminar black phosphorus. It is the layered material depicted in Figure 2f, and this material can undergo exfoliation to form 2D nanomaterials. However, the exfoliated nanosheet material is highly unstable in water and air, as it oxidizes readily. To overcome oxidation, the exfoliation of nanosheets in the presence of NaOH enables the attachment of -OH groups on the surface and ensures stability in water [66]. The selective functionalization of nanosheet edges is also known to stabilize black phosphorus under ambient conditions [67].

Stable nanosheets are paramount for their intended use as building blocks. The lack of stability concerns not only BP, but also certain metal organic frameworks (MOF) [68]. This term refers to a large group of nanostructures, which includes nanosheets and nanoparticles. Although interest in nanosheets has increased over the years, and reviews discuss their fabrication as well as application, nanoparticles are predominant and mostly used for membrane fabrication [69,70]. The general molecular structure of MOF nanosheets is described by metal ions and organic ligands coordinated in a plane that forms regular pore arrays. The definition of MOF is broad, and it includes a large number of crystal structures with different functionalities [71].

Another group with a wide variety of crystal structures and applications are so-called layered double hydroxides. LDH nanosheets are considered to be a type of clay and they are defined with the molecular formula, $\begin{array}{c} {}[M_{1-x^{2+}}M_{x^{3+}}(OH{)}_{2}][A^{n-}{]}_{x/n}\cdot mH_{2}O \end{array}$. Divalent metal ions Mg^2+^, Zn^2+^, Cu^2+^ or Ni^2+^, trivalent metal ions Al^3+^, Fe^3+^, Ga^3+^, or Mn^3+^, and charge compensating anions $\begin{array}{c} CO_{3^{2-}} \end{array}$, NO^3−^, $\begin{array}{c} SO_{4^{2-}} \end{array}$, and stearate, are represented by M^2+^, M^3+^, or A^n−^, respectively [72]. LDH crystals have either rhombohedral or hexagonal structures, with brucite-like layers that are separated by charge compensation as well as solvation molecules. Furthermore, the metal ions are present in the center of the structure, whereas the edges are occupied by hydroxide ions interconnecting octahedra to form nanosheets [73].

In comparison to the complex structure of LDHs, boron nitride nanosheets share similarities with carbon-based materials. They form a regular mesh of nitrogen and boron honeycombs. There are three molecular structures: hexagonal h-BN, rhombohedral r-BN, and cubic c-BN. Especially, hexagonal boron nitride receives much attention in the field of membrane science [74]. Despite the similar structures of graphene and BN, they differ in their properties as conductors and insulators, respectively [75].

### 2.3. Types of Nanosheet Membranes

**Figure 3:**
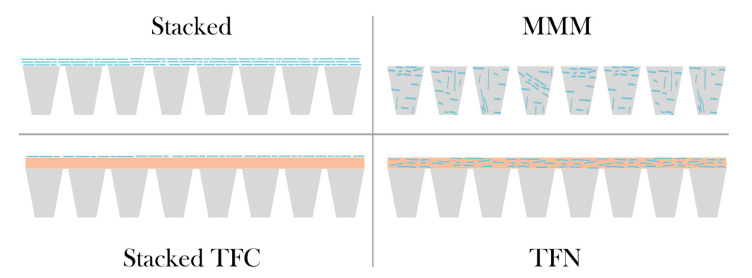
The types of nanosheet membranes that demonstrate improved membrane performance in terms of fouling mitigation. Other types of nanosheet membranes have the nanosheets dispersed in the support layer of a Thin Film Composite (TFC) membrane, or consist of a porous monolayer nanosheet. The illustration depicts as ideal porous polymer substrate with a cross-sectional view. Blue rectangles represent nanosheets that are either immersed in the grey polymer support and the orange PA layer, or stacked on top of the polymer and the PA.

Nanosheet membranes can be divided into blended, stacked, or monolayered versions. The separation of ions with single-layered nanosheets is achieved using materials with intrinsic porous structures or by forming pores. To date, large-area pore drilling and uniform pore formation are challenges that are addressed by researchers [27]. Although nanoporous membranes exhibit promising features, further studies are required to fully understand the fabrication methods and subsequently allow scale-up [76]. In terms of nanocomposites, the synthesis of membranes can be achieved either by embedding the nanostructures in a polymer or by coating or grafting approaches with nanosheets on a support. Figure 3 illustrates nanocomposite membranes that are based on 2D nanosheets. The blending of nanosheets into the substrate yields so-called Mixed Matrix Membranes (MMM), where the nanostructures are dispersed in a dope solution during fabrication [77]. Another approach is the immersion of nanostructures in the polyamide top layer of Thin Film Composite (TFC) membranes. However, in this case, it would be considered to be a Thin Film Nanocomposite (TFN) membrane [78,79]. For stacked nanosheet membranes, nanosheets are either deposited or grafted directly onto the support or polyamide layer. Herein, we discuss MMM and surface functionalized membranes. Information regarding nanoporous monolayer nanosheet membranes can be found elsewhere [27].

#### 2.3.1. Stacked Nanosheet Membranes

**Figure 4:**
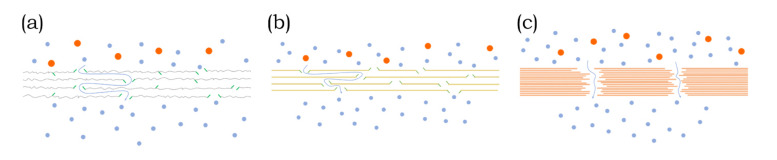
Stacking structure of laminar membranes that reject pollutants and yet allow water to pass, illustrated as red and blue circles, respectively. The water pathways are indicated by blue arrows. (**a**) Corrugated GO membrane with functional groups highlighted in green and located at the nanosheet (grey) edges and on the surface. (**b**) Transition Metal Dichalcogenides (TMD) membrane with functional groups highlighted in green are located at the nanosheet (yellowish) edges and in the rigid nanostructure. (**c**) Niobate membranes with small interlayer spacing and vertical separation due to pore formation.

Coating with nanosheet nanofilms is achieved while using a method from one of the following five main categories: dip- [80], spin- [81], spray- [82], electromagnetic coating [80], vacuum- or pressure-assisted filtration [83,84], and the layer-by-layer approach [85]. Coating with nanosheets forms a selective barrier that permits water permeation, yet restricts the passage of other molecules. As a result, the presence of nanosheets on the surface alters the separation mechanism of the pristine membrane, which is then governed by size exclusion and electrostatic repulsion. In contrast to polymeric and ceramic membranes, diffusion is limited [86,87]. The thickness of the membrane skin layer is responsible for the separation and rejection of molecules present in the feed stream. Thus, an increase in the nanosheet layer results in a decrease in permeance. In this regard, nanosheet membranes offer a unique opportunity to directly control the thickness of the selective barrier. This is achieved by controlling the concentration of nanomaterials in the feed solution during the vacuum filtration membrane assembly [86]. Theoretically, only a few layers of nanosheets are required in order to completely cover the substrate and form a selective layer for effective membrane separation. However, defects and random stacking can result in the need for several layers to ensure separation. In contrast to polymeric membranes that feature a wide distribution of pore sizes, stacked nanosheet membranes allow for precise sieving due to a narrow nanochannel distribution [88]. The 2D laminar membranes exhibit different membrane structures depending on the nanosheet material, as illustrated in Figure 4. Subfigures a and b show horizontal nanochannels formed between stacked nanosheets. Vertical nanochannels appear due to pores in the nanosheets as well as between the edges of nanosheets. In contrast, subfigure c only consists of vertical nanochannels.

GO is a highly flexible material that forms nanowrinkles and hydrophobic interlayer nanochannels with hydrophilic pores. This structure causes electrostatic repulsion of substances, such as salt ions, while simultaneously enhancing the water flux [89]. Upon hydration, hydroxyl groups on the surface of GO partially deprotonate and induce electrostatic repulsion due to the negative charge, which consequently results in a redispersion of the nanosheets in water. To overcome the instability of GO in water, either crosslinkers, such as cationic species Al^3+^ and Mn^2+^ and amine molecules, are employed [90,91]. Alternatively, GO is reduced to rGO by removing the groups attached to the nanosheet surface [92]. The charged nanosheet edges cause the membrane structure to avoid edge-over-edge configurations and form nanowrinkles, which. In combination with irregular stacking, this benefits water transport by forming numerous nanochannels [93]. Removing the hydrogen-bonded hydrophilic groups from GO smooths the rGO [94]. To increase water flux by modifying the nanochannels, methods, such as thermal treatment, are employed [95] in order to corrugate the nanosheets. Additionally, the intercalation of nanomaterials increases the interlayer distance [96,97] and reduces GO. Large GO sheets form longer hydrophobic nanochannels that enable water to pass faster and thus increase the flux. The flexibility of graphene oxide nanosheets causes linear compaction upon pressure application. This effect allows a tuning of the separation performance that either increases or decreases the interlayer spacing in response to pressure [86]. Despite the availability of solutions to alleviate the instability of GO, these approaches are too complex to scale up [98]. Hence, other nanosheet materials are promising alternatives for the replacement of GO.

TMDs are promising alternatives to GBN, as they form stable laminar layers in water due to strong Van der Waals forces and the absence of functional groups extruding from the surface [99]. Although crosslinkers are not required for TMD nanosheet membranes to retain their stability, they irreversibly restack under dry conditions. Even in cross-flow conditions, no removal of nanosheets was observed, whereas non-crosslinked GO peeled off from the substrate at shear stresses as low as 0.2 N m^−1^. Despite their stability in water without additional cross-linking, MoS_2_ functionalization with dye was reported to increase salt rejection and maintain high levels of permeance. Hirunpinyo-pas et al. [100] reported the fabrication of stacked MoS_2_ nanosheets membranes functionalized with dyes, that retained about 99% ions found in sea water and, thus, achieved drinking water quality. Another advantage of this group of materials is that MoS_2_ and WS_2_ membranes have demonstrated stable interlayer tuning properties by compaction upon pressure applications. In this case, the interlayer distance was retained, even after the release of pressure [101]. These nanosheets have a high degree of surface smoothness, which can be explained by the lack of functional groups and crosslinkers. The resultant low hydraulic friction promotes high water flux in the nanochannels [102]. Although the basal planes of MoS_2_ and WS_2_ are free of functional groups, their entire surface is hydrophilic, because of the evenly distributed sulfur atoms on both sides of the nanosheet. Another advantage of TMDs is a chemical structure that is comprised of a three atomic monolayer molecular composition that gives them rigidity [99].

MXene forms another group of nanosheets that suffers from swelling with water when a nanosheet layer is stakced on top of a substrate. However, Lu et al. [103] reported successful self-crosslinking of nanosheets via dehydroxylation on the surface of the basal planes. Strengthening the membrane structure via self-crosslinking resulted in the long-term stability of ion rejection, even under harsh acid or basic environments. Another approach for mitigating swelling is the intercalation of ions that interact with the oxygen-containing functional groups to increase stability [104]. Additionally, stacked Ti_3_C_2_T_*x*_ nanosheet membranes demonstrated ultrafast water flux with precise sieving performance and high selectivity for cations [57].

MOF nanosheet membranes are also considered to be unstable in water and require crosslinkers in order to retain their structure in water. In the field of MOF nanosheet membranes, most attention has been given to gas separation rather than to water purification [105].

Among TMO nanosheets, niobate membranes are reported to have a structure that differs from that of other laminar membranes [106]. The acid sites on the surface of the niobate nanosheets [48,107] are crosslinked with triethanolamine (TEOA) and form layered structures with a free spacing of 0.2 nm [108]. Under wet conditions, the layered structure remains the same and inhibits molecular sieving via interlayer spacing. Therefore, vertical void structures were considered to be nanochannels that account for the separation mechanism of these laminar membranes [109].

#### 2.3.2. Conventional Mixed Matrix Membranes

Organic membranes blended with inorganic fillers for water purification applications often employ PES, PEI, PAN, PSf, and PVDF polymers. Non-Solvent Induced Phase Separation (NIPS) is a common synthesis route for the respective membranes [29,110]. This is recognized as an approach to the fabrication of nanocomposite membranes and it is easily implemented in existing production lines by blending a dope solution and nanosheets [111]. Nanostructured materials are either added to the substrate to form conventional nanocomposite membranes, or to the thin film on the top layer on a substrate to obtain TFN. The incorporation of nanosheets can improve the overall hydrophilicity, surface roughness, and permeance of the membranes [112].

**Figure 5:**
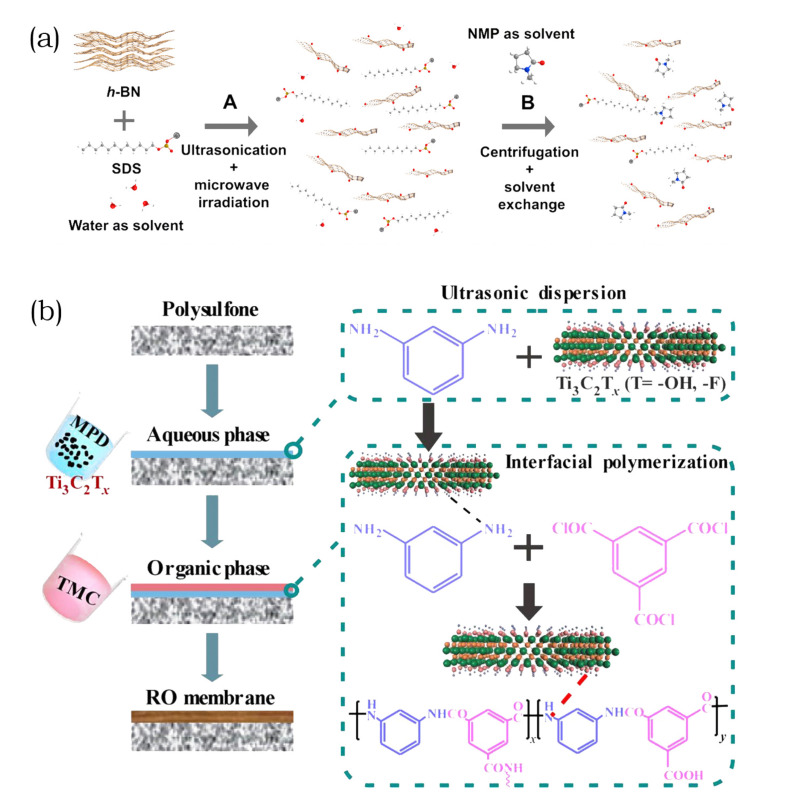
(**a**) The exfoliation process of boron nitride nanosheets from their bulk counterparts. Reprinted from [29], Copyright (2018), with permission from Elsevier. (**b**) Fabrication steps of Ti_3_C_2_T_*x*_ Thin Film Nanocomposite (TFN) membranes via interfacial polymerization of m-phenylenediamine (MPD) and trimesoyl chloride (TMC) on a PSf support. Reprinted from [30], Copyright (2020), with permission from Elsevier.

For conventional nanocomposite membranes, nanosheets are added to polymers to fabricate pressure-driven MF, UF, and NF membranes. These membranes are classified based on their pore size as well as their molecular weight cut off (MWCO), with a separation mechanism that is based on size exclusion. However, NF membranes can show a mix of size exclusion and diffusion [113]. The addition of hydrophilic nanosheets improves membrane performance by increasing the porosity, the elongation, and the widening of microvoid fingers. The affinity of hydrophilic nanosheets to hydrophilic groups in a polymer solution increases the mass transfer rate between solvents and nonsolvents during the fabrication process, which results in the elevation of membrane porosity. By affecting the thermodynamic instability, during phase separation, the number and size of pores are increased. Induced instability is the reason for accelerated liquid-liquid phase separation [110,114]. The optimal concentration of nanosheets in the blend solution must be determined, as an excess of fillers increases the viscosity and subsequently reduces the pore size [115]. Excessive addition of nanosheets to the dope solution delays mixing separation, and results in a top layer that is both denser and thicker with less pore connectivity. When using BN as a nanofiller for a conventional MMM, the synthesis route via sonification is shown in Figure 5a. With the addition of BN, the pore structure becomes finger-like and more connected, whereas at a wt% of 0.05, pores undergo unfavorable changes in structure [29]. Another reason for enhanced permeance due to the presence of hydrophilic nanosheets is their migration towards the membrane surface. During the fabrication, nanosheets migrate to the surface in order to reduce the interface energy [116]. When the nanosheet concentration passes the optimized level, it can lead to a decrease in hydrophilicity and a simultaneous increase in surface roughness due to aggregation [30,117]. For example, GO has low solubility in organic solvents and causes agglomeration in bulk solutions [114,118]. Functionalizing nanosheets and the formation of heterostructures with other nanomaterials are methods that can be used to improve their dispersion in a polymer. Additionally, it improves the compatibility of nanofillers and a membrane substrate [77,118,119]. However, when physically blended, nanomaterials sometimes show good dispersion within a membrane. Nonetheless, the majority could be embedded in the bulk polymer, which lowers the modification efficiency [120]. Xu et al. [121] and Huang et al. [122] addressed the limited exposure of nanosheets on a membrane surface while applying a magnetic field during the phase-inversion process. Consequently, migration behavior and alignment were improved.

#### 2.3.3. TFC and TFN Membranes

TFC membranes are characterized by a thin selective layer on a porous substrate that provides mechanical stability. This design renders TFC membranes popular for NF, RO, and FO applications. The governing separation mechanism for such membranes is the solution-diffusion model, where water and solutes dissolve in the dense PA film and diffuse through the membrane. The same film that permits the diffusion of water and solutes retains other molecules [123]. Water permeance through PA TFC depends on the degree of hydrophilicity, and on the thickness and cross-linking of the thin film. Lowering the degree of cross-linking to improve water flux is achieved by incorporating hydrophilic nanosheets that disturb the reactions during thin film formation. Additional effects that improve the performance of such nanocomposite membranes include the formation of nanochannels and changes in the surface properties in terms of charge density. When nanosheets are added to PA precursors MPD and TMC during interfacial polymerization (IP), TFN membranes are formed, as demonstrated in Figure 5b [112]. Although nanosheets remain in the bulk polymer, similar to conventional MMM, the higher density of fillers as compared with that of water limits the surface exposure of the PA layer in TFC membranes. Due to the stability of GO in water, they exhibited a better dispersion in PA. The presence of MXene, functionalized GO, and GO/CNTs was reported to increase the resistance of the otherwise sensitive polyamide layer to chlorine by acting as radical scavengers [77,124].

## 3. Antifouling Nanosheet Membranes

### 3.1. Fouling Mechanism

This chapter provides only a brief explanation of the fouling mechanisms, because this topic has already been broadly discussed in many publications. Fouling involves several mechanisms and it s considered a bottleneck in membrane applications. Different mechanisms are predominantk, depending on the type of foulant and membrane operation. Fouling in microfiltration (MF), ultrafiltration (UF), nanofiltration (NF), and reverse osmosis (RO) can be described as the total membrane resistance ($R_{tot}$), as characterized by Equation (1).

(1) $$R_{tot}=R_{m}+R_{c}+R_{p}+R_{a}+R_{cp}$$

The total membrane resistance ($R_{tot}$) includes the hydraulic resistance ($R_{m}$) of the membrane itself and four fouling mechanisms: cake layer formation ($R_{c}$), pore blocking ($R_{p}$), adsorption ($R_{a}$), and concentration polarization ($R_{cp}$). The fouling resistance, $R_{f}$, is obtained by subtracting $R_{m}$ from $R_{tot}$. In addition, membrane fouling is also divided into reversible ($R_{r}$) and irreversible ($R_{ir}$) fouling, as shown in Equation (2). The former is alleviated by physical cleaning to remove cake layers and by concentration polarization. The latter remains after washing with water, but can be removed via chemical cleaning.

(2) $$R_{f}=R_{r}+R_{ir}$$

The fouling behaviour for forward osmosis (FO) differs from that seen in previous membranes. In general, the fouling propensity for FO is lower when compared with other membranes, which can be explained by a lower level of hydraulic pressure. Additionally, fouling in FO is almost completely reversible [125]. For a better understanding of FO fouling mechanisms, please refer to previously published studies [126,127].

The types of foulants can be classified into three groups, depending on the foulant material: organic, inorganic, and biofouling. Furthermore, these foulants show characteristic fouling behaviors, and organic fouling can be subdivided into either spreadable or non-migratory foulants. The former is comprised of oils that form a continuous layer. By contrast, the latter includes natural organic matter (NOM) as well as biomacromolecules that adhere to the membrane surface [19]. Non-migratory foulants cause cake-layer formation [128], foulant deposition in pores, or adsorption on the surface. Forces contributing to protein fouling include hydrogen bonding, electrostatic attraction, and Van der Waals forces [129]. Because conventional water treatment technologies are incapable of removing oil-surfactant emulsions, fouling resistance of membranes to oils is of increasing importance. Therefore, membranes are a viable solution that nonetheless suffers from oil film formation that blocks the passgae of water [130]. Bio- or proliferative foulants encompass microorganisms that adhere to the membrane surface. They continuously deteriorate the separation performance via reproduction and growth of a biofilm. Although this type of fouling is equally important to organic and inorganic fouling, it will not be covered in this review. For more information, it is necessary to consult previously published studies [19,33,131,132,133,134,135,136,137].

The last category of foulants describes inorganic fouling, also called scaling, and it includes the precipitation of salts on the membrane surface due to concentration polarization. The accumulation of inorganic material on the membrane surface results in an increase in concentration beyond the solubility of these materials, which then precipitates. The term scaling is used when the precipitated salts form a scale on the membrane surface. Two types of scaling mechanisms have been defined. Heterogeneous scaling describes nucleation on the membrane surface. Homogeneous scaling refers to the nucleation of a bulk substance within a liquid. Although the effect of each mechanism on the total scaling of a membrane is still being investigated, it is suggested that heterogeneous scaling plays a major role, as it has a lower energy barrier to overcome [138]. The crystallization of salts is directly dependent on pH, temperature, and flow velocity. In terms of membrane antifouling strategies, inorganic fouling is addressed with commonly applied technologies. These include the pre-treatment of the feed via chemical softening or multistage filtration, and the use of antiscalants to retard further crystal growth. Alternatively, improving the process operation alleviates inorganic fouling via pH adjustment, backflushing, and flow, as well as temperature changes [138,139].

Another class of foulants is the so-called colloidal or particulate fouling that comprises inorganic and organic matter. Their characteristic fouling behavior includes pore blocking and cake layer formation. While inorganic colloids might result from homogeneous scaling and form cake layers, organic colloids can adsorb to the membrane surface. Therefore, the type of colloid material and size governs the fouling mechanism and the respective strategy to alleviate fouling [12].

### 3.2. Antifouling Strategies

**Figure 6:**
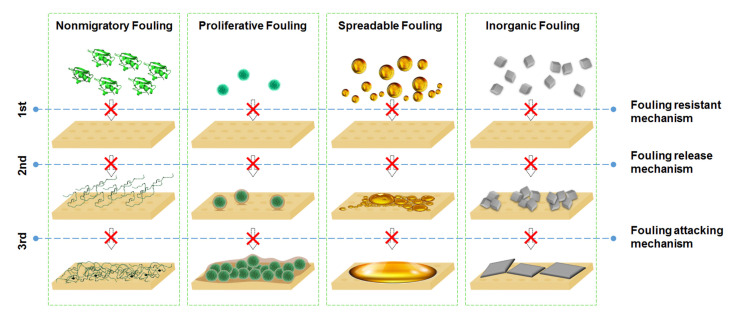
The passive and active antifouling strategies for non-migratory, spreadable, proliferative and inorganic foulants. Reprinted from [133], Copyright (2018) with permission from Elsevier.

Zhang et al. [19] classified the antifouling membrane modification strategies as either passive or active. Passive strategies rely on membrane surface modifications to hamper foulant adhesion and facilitate removal. These strategies include fouling resistance that is achieved by developing a hydrophilic surface. Fouling release involves the formation of amphiphilic surfaces that drive the foulants away from the membrane. By contrast, active antifouling strategies focus on destructing the foulant via membrane surface contact or agent release. These categorizations of antifouling strategies for membranes were previously described by Zhang et al. [19], and they are illustrated in Figure 6. In this review, the application of nanosheets for passive strategies such as improving hydrophilicity, surface roughness and surface charge will be discussed. Although nanosheets impart bactericidal properties, herein only the photocatalytic activities of 2D nanomaterials will be discussed.

#### 3.2.1. Hydrophilicity

Membrane modification to render surfaces fouling resistant without destructing the foulants is a bordly applied technique to improve filtration performance and prolong membrane lifetime. Functionalization with nanomaterials as fillers or surface modifiers changes the surface charge, topography, and pore size, as well as the distribution, hydrophilicity, and wetting behavior [129]. Common antifouling modifications include polymer brushes, zwitterions, and superhydrophilic nanomaterials.

Water contact angle measurements are conducted in order to determine the hydrophilicity or hydrophobicity of a membrane, and the results are applied to Young’s equation (Equation (3)). It describes the thermodynamic equilibrium of a liquid, a vapor, and ideal solid surfaces defined as inert, homogeneous, rigid, and smooth [140].

(3) $$\gamma_{SV}=\gamma_{SL}-\gamma_{LV}\;\cdot \;\cos\left(\theta \right)$$

In Equation (3), γ values represent the interfacial energies between the phases, whereas θ is the contact angle that was obtained via measurements. In terms of interfacial energies, $\gamma_{SV}$, $\gamma_{SL}$, and $\gamma_{LV}$ represent solid-vapor, solid-liquid, and liquid-vapor interfaces, respectively. The degree of the water contact angle defines whether a material is hydrophilic (θ < 90°), superhydrophilic (θ < 10°), hydrophobic (θ > 90°), or superhydrophobic (θ > 145°) [140,141]. In terms of hydrophilic membranes, water molecules are adsorbed on the surface, which weakens binding to other molecules, such as organic pollutants. However, hydrophilic membranes are only fouling resistant to hydrophilic material. By contrast, hydrophobic surfaces are only resistant to hydrophobic substances [142].

Although hydrophilic membrane materials are known to reduce fouling, hydrophobic polymers are predominantly employed due to their stability in aqueous solutions [143]. Common hydrophilic polymers that find application in water treatment include cellulose and its derivatives, PES, PSf, PC, PA, and PAN. By contrast, hydrophobic materials comprise PTFE, PVDF, and polyethylene. Researchers address the stability-fouling trade-off by hydrophilizing otherwise hydrophobic membranes. Such modification methods feature zwitterions or brushes, PDA coatings, and the use of nanomaterials [133]. The resultant membranes are resistant to fouling of hydrophobic NOM, proteins, and oils. The relevant foulants for water purification are mostly hydrophilic and, thus, repelled by hydrophilic membranes. Nonetheless, certain hydrophobic water constituents including NOM and biopolymers are repelled from hydrophobic membranes, yet cause in fouling of hydrophilic membranes [144].

The Wenzel [145] and Cassie-Baxter [146] models describe the wettability of rough surfaces. For the Wenzel model, surface roughness intensifies the hydrophilicity due to a higher level of net surface energy that decreases during wetting. Hydrophobic rough surfaces have an even lower level of surface energy, which is a stronger repellent of water due to an unfavorable increase in energy upon wetting. The Wenzel model describes increases in both hydrophobicity and hydrophilicity due to surface roughness. Enhanced hydrophilicity results from the replacement of solid-air interfaces with an equal area of liquid-solid interfaces. In contrast, the Cassie–Baxter model suggests the formation of air pockets, which results in the presence of both solid-liquid and solid-air interfaces in the water droplets on solid surfaces [140]. The Miwa-Hasimoto model is a combination of both models [147].

The creation of a surface that is both superhydrophilic and superoleophobic is difficult, because materials that repel oil would also repel water, which has a higher level of surface tension. The mechanism of such membranes is based on the presence of dispersive and non-dispersive forms of free surface energy such as Van der Waals and hydrogen bonding, respectively. In their work, Pan et al. [148] found that coexisting superhydrophilic/superoleophobic membranes have a high concentration of non-dispersive surface free energy and low-dispersive components. Wang et al. [149] reported a phenomenon of photo-induced amphiphilicity for TiO_2_ after the otherwise completely hydrophobic surface was turned hydrophilic by UV irradiation. Underwater superhydrophilicity of TiO_2_ is assumed to be the result of stronger water bonding during light irradiation [150]. Surface free energy is also susceptible to changes via other stimuli, such as heat and electrical fields [151,152]. Heterogeneous surface structures enable the formation of a hydration layer that prevents foulants from adhering to the surface. Simultaneously, heterogeneous surfaces enhance the removal of foulants with low surface energy due to the low surface tension compartments [153,154]. Materials that contain fluorine and silicon are commonly applied to create structures with low surface free energy to alleviate fouling, which consequently reduces permeance [155,156]. Superhydrophilicity is beneficial in terms of the prevention of spreadable fouling, but it suffers from aggravated cleaning due to the very strong adhesion of foulants that are able to pass the hydration layer [111]. Generally, membranes that allow water to permeate while repelling oil posses amphiphilic properties that are based on three parameters. These include surface roughness, selectively high surface energy, and non-uniform surface chemistry [111].

Different approaches are employed to increase hydrophilicity: surface charges, energy barriers, and alteration of surface roughness. These approaches are achieved by membrane modifications via zwitterions, polymer brushes and nanomaterials.As discussed in the previous chapter, a conventional MMM successfully addresses the permeability-selectivity trade off by increasing water flux without sacrificing rejection rates. Changes in pore structure and hydrophilicity are the reason for these favorable changes in membrane performance. Surface roughness can be decreased via the formation of a dense and smooth top layer in a MMM. By contrast, coated or grafted nanosheet membranes show improved hydrophilicity as compared with their pristine support due to direct exposure of the nanomaterials to the feed. An alternative to the membrane functionalization by employing nanomaterials is the application of zwitterions that are either coated, grafted onto, or blended with the matrix [157]. Both zwitterions and hydrophilic nanosheet modified membranes form a hydration layer as a physical and energetic barrier for foulant adsorption. However, the hydration layer of hydrophilic surfaces is characterized by hydrogen bonds. However, surfaces modified with zwitterions form strong electrostatic bonds with water due to both negative and positive charges. Because ionic bonds are stronger, more energy is required in order to replace water molecules with foulants. By contrast, hydrogen bonds are weaker and, thus, subject to the expulsion of water and the adhesion of proteins [158]. A rather new approach to render membranes fouling resistant is the modification of nanofillers with zwitterions. As a result, both the agglomeration of GO in the cast solution and the miscibility of zwitterions are improved, so that the membrane demonstrates a smoother surface and higher hydrophilicity [159,160].

#### 3.2.2. Photocatalysis

**Figure 7:**
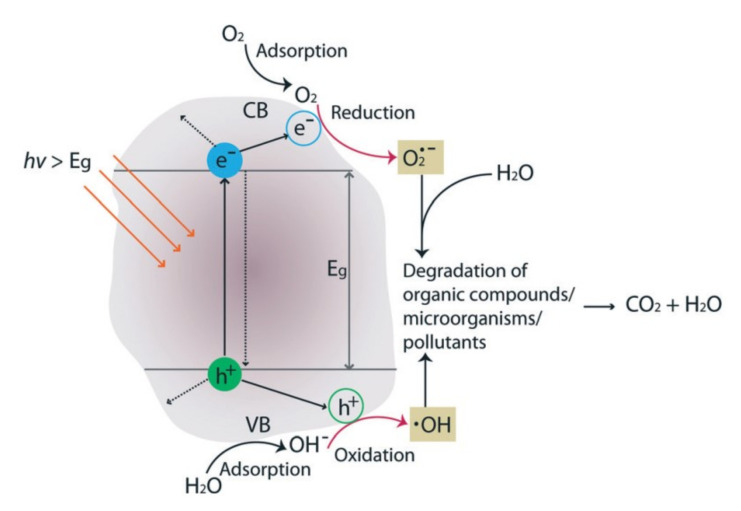
The photocatalytic effect of a catalyst upon light irradiation, including the formation of reactive oxygen species (ROS) and final products. Reproduced from [166] with permission from The Royal Society of Chemistry, Copyright (2016).

The application of nanostructures has gained popularity, because of their intrinsic photocatalytic properties. Such semiconductor materials are characterized by a valence band (VB) (+1.0 to +3.5 V vs. NHE) filled with electrons and by an empty conduction band (CB) (+0.5 to −1.5 V vs. NHE) [161]. Upon light irradiation, excited photons (hν) that exceed or match the band gap width ($E_{g}$) induce electron ($e^{-}$) transfer to the CB, which creates holes ($h^{+}$) on the VB. Usually, electrons and holes recombine quickly while releasing heat. Unless they either react with electron acceptors or donors on the membrane surface or they are trapped in their state. Electrons in the CB are reductants that form radicals, such as superoxide and ${}^{\cdot }OOH$, in the presence of oxygen. Whereas holes in the VB are oxidants that form hydroxyl radicals in water as shown in Figure 7. Hence, irradiated semiconductors can be employed to degrade pollutants that are adsorbed to semiconductors directly at the $h^{+}$ oxidation sites, or via the radicals as shown in Equations (4)–(8) [162,163]. The pollutants are degraded via intermediates to the final products H_2_O, CO_2_ and inorganic acids. Malato et al. [161] summarized the parameters that influence photocatalysis as initial reactant concentration, the catalyst mass, the oxygen concentration, the temperature, the pH, and the radiant flux.

(4) $$\mathrm{Photocatalyst}+h\nu ⟶e^{-}+h^{+}$$

(5) $$h^{+}+H_{2}O⟶{}^{\cdot }OH+H^{+}$$

(6) $$h^{+}+OH^{-}⟶\cdot OH$$

(7) $$O_{2}+e^{-}⟶O_{2}^{\cdot -}$$

(8) $$O_{2}^{\cdot -}+H^{+}⟶\cdot OOH$$

In addition to the generation of reactive oxygen species (ROS) upon light irradiation, certain semiconductors also demonstrate an altered wettability. Wang et al. [149] were the first to report a change in the water contact angle of TiO_2_ anatase films of 72 ± 1${}^{{}^{\circ}}$ to 0 ${}^{{}^{\circ}}$ following light irradiation. This effect can be explained by the reductions in the Ti state of oxygen vacancies that improve water affinity [140]. Simultaneous light-induced oxidation and hydrophilic properties were also observed for the rutile phases of TiO_2_, ZnO, SnO_2_, TiNbO_5_, Ti_2_NbO_7_, Ti_5_NbO_14_, and Nb_3_O_8_ [164,165]. Miyauchi et al. [164] reported photoinduced hydrophilicity without photocatalytic oxidation for WO_3_ and V_2_O_5_.

More specifically, the photocatalytic activity of semiconductors also depends on their characteristic light absorption spectra and band gap width. In the case of TiO_2_, the band gaps for the rutile and anatase phases are 3.0 eV and 3.2 eV, respectively, which limits photoactivity to the UV region [140]. TMO, TMD, and MXene, are photosensitive materials that are employed in catalysts, such as TiO_2_ [167], ZnO [168], Fe_2_O_3_ [169], Nb_3_O_8_ [170], WO_3_ [171], and MoS_2_ [172]. Despite their promising features, semiconducting materials are still subject to ongoing investigations in order to improve their photocatalytic performance. Jo et al. [25] summarized the opportunities for catalyst improvement: (i) change of adsorption spectra, (ii) inhibition of charge recombination (iii) faster transport of charges to the bulk surface, and (iv) an increase in surface reaction sites. The solutions for these shortcomings are doping of the semiconductor with non-metal or metal materials, co-doping with metal-non-metal compounds, dye sensitization, and the formation of heterojunctions [140].

The advantage of 2D photocatalysts is an atomic thickness that exposes almost all the ions to the surface and causes the formation of strong electronic coupling with other materials. In contrast to doped semiconductors, nanohybrids show no changes in their material structure. In particular, 2D nanomaterials are widely employed as photocatalysts, either alone or as co-catalysts, in order to address the drawbacks of the semiconductors currently being used. Heterogeneous structures effectively alleviate problem (i) and (ii) by changing the band gap width and transporting electrons as well as holes to suppress their recombination. Problems (iii) and (iv) are addressed by employing the high-aspect ratio of nanosheets as building blocks for hybrid photocatalysts. That reduces the time that is required for electrons and holes to migrate to the surface where the large lateral size provides many reaction sites [25]. The increased interest in nanomaterials and research output has resulted in an infinite number of photocatalytic nanohybrids. In most studies, the focus lies in the photocatalytic activity of nanostructures to degrade organic pollutants and CEC [173,174,175,176,177]. Nevertheless, another promising benefit of photocatalysis relevant for water purification is the possible reduction of heavy metals. Heavy metals pose a threat to the human health when consumed and can be detected in industrial waters and natural water resources. Their reduction via photocatalysis was reported by several groups, such as Liu et al. [178], who employed porous BN/TiO_2_ nanosheets. Other studies demonstrated the removal of radioactive compounds via adsorption on nanocomposites [179].

Integrated photocatalytic membrane processes combine the catalytic degradation of pollutants with conventional water purification via separation. Photocatalytic membrane reactors (PMR) are recognized to improve separation in general. Nonetheless, they are known to also remove CEC and other harmful substances from water that are otherwise present in the permeate [180,181]. Photocatalysts in PMR are either immobilized on membranes or suspended in the feed solution [182,183]. Immobilized PMRs are comprised mostly, or exclusively, either of NP semiconductors or GO-doped NP [184,185]. Studies into the important roles and advantages of nanosheets as photocatalysts are increasing, as shown by the reviews that are listed in our references [25,186]. However, photo-responsive nanosheet membranes are seldom studied for both their separation and photocatalytic performance. Thus far, the nanosheet materials employed for photocatalytic membranes have been limited mainly to GO, rGO, gCN(H), and TiO_2_. The two previously mentioned examples of GBN act as building blocks for the catalyst TiO_2_ and are the most frequently reported nanohybrids used in integrated water treatment [187,188,189]. More recently, gCN(H) functionalized membranes have become a popular alternative to either replace TiO_2_ as photocatalyst, or by forming heterostructures with other nanomaterials [190,191,192]. Undoubtedly, TiO_2_ is the most intensively studied photocatalyst that is usually employed as a NP, but some studies have reported the performance of nanosheets [193,194]. Several other 2D nanocomposites are known to possess photoactivity, yet only a handful of different nanosheets are have been employed as building blocks for photocatalytic membranes [195]. Although the interest in 2D nanocomposite photocatalytic membranes has increased, their antifouling propensity has been explored less. Therefore, photocatalytic nanosheet membranes will also be discussed in the following sections.

## 4. Nanosheet Induced Fouling Mitigation

### 4.1. Non-Migratory Fouling Strategies

The vast majority of antifouling nanosheet membranes employ GO, rGO, and their functionalized derivatives. However, in this work, a selection of publications is summarized to demonstrate the diversity of antifouling nanosheet membranes. In terms of non-migratory fouling, mostly BSA, MB, HA, and SA have been employed as model foulants under dark conditions as listed in Table 1. For the less intensively investigated photocatalytic membranes, mostly organic dyes have been used for testing as shown in Table 2. The roles of nanosheets in terms of non-migratory fouling mitigation have involved photocatalytic foulant degradation, surface modification to improve surface energy, hydrophilicity and roughness, and grafting sites for zwitterions or brushes.

Alam et al. [196] analyzed GO, rGO, and MoS_2_ stacked on a PES support. GO membranes had the lowest water contact angle at 40 ± 1.12${}^{{}^{\circ}}$ and together with rGO uneven surfaces. While MoS_2_ with a contact angle above 50${}^{{}^{\circ}}$, exhibited high surface smoothness due to the rigid structure of the nanosheets. MoS_2_ membranes had the highest level of water permeability, and both MoS_2_ and rGO membranes revealed a small compaction response to pressure applications. All of the membranes rejected BSA and SA molecules, even when the pore diameter was larger than the molecules. This effect is the result of the repulsion between the negative surface charges of membranes and molecules, and the formation of a hydration layer on the membrane surface. Fouling studies have revealed fouling recovery rates for MoS_2_/PES that were higher than those for other membranes, suggesting that washing for flux recovery is less frequently required. By comparison, MoS_2_, GO, and rGO experienced 43, 17, and 6% reversible fouling. The low value for rGO was the result of the hydrophobic nature of the nanosheets, which hampered the release of foulants. Whereas, the lack of functional groups on MoS_2_ was the reason for low levels of foulant adsorption. Despite different properties, all of the modified membranes showed improved foulant rejection and less of a fouling propensity compared with that of pristine PES membranes [196].

**Table 1:** Nanosheet membranes to mitigate non-migratory fouling.

Nanosheet	Type	Materials	Application	Foulant	WCA	Highlights	Ref.
GO	Stacked	PDDA, PAN(S)	NF	BSA, HA, SA	∼50${}^{{}^{\circ}}$, increases with layers	LbL fabrication, FRR-HA = 91.2%, FRR-BSA = 92.7%	[197]
GO, rGO or MoS_2_	Stacked	PES(S)	NF	BSA, SA	GO 40 ± 1.12${}^{{}^{\circ}}$	MoS_2_ has highest flux and FRR	[196]
PMSA-GO	MMM	PVDF(S)	NF	BSA		zwitterions incorporated, better dispersion of GO, FRR = 95.3%	[160]
WS_2_	Stacked	AAO(S)	NF	BSA	92.6${}^{{}^{\circ}}$	FRR = 74.04%	[98]
WS_2_	MMM	CA(S)	UF	BSA	63.3 ± 1.6 ${}^{{}^{\circ}}$	FRR = 99.2 ± 0.8%	[198]
MoS_2_	Stacked	PEI, PAA, PES(S)	FO	BSA	<90${}^{{}^{\circ}}$	LbL fabrication	[85]
MoS_2_ or GO	MMM	PAI(S)	UF	HA, BSA	lower for MoS_2_	higher FRR for MoS_2_	[199]
MMT or LDH	TFN	PA(TF), PSf(S)	RO	BSA, DTAB, TA	MMT = 47.2 ${}^{{}^{\circ}}$, LDH = 52.3 ${}^{{}^{\circ}}$	different fouling behaviour because of nanosheet surface charge	[200]
gCN(H)	TFN	PDA(C), PA(TF), PES(S)	NF	BSA	>60${}^{{}^{\circ}}$	FRR > 95%	[201]
gCN(H), rGO	Stacked	TiO_2_-NP, PVDF(S)	UF	BSA	18 ± 8 ${}^{{}^{\circ}}$	FRR = 86.1%	[202]
BN	MMM	PES(S)	NF	HA	56 ± 2${}^{{}^{\circ}}$	complete flux recovery	[29]
A-BN	Stacked TFC	PPA(TF), PES(S)	NF	SA, BSA	25 ± 0.33${}^{{}^{\circ}}$	R_ir_−SA = 2.1 ± 0.3% and R_ir_−BSA = 7.0 ± 2.0%	[203]
Ti_3_C_2_T_*x*_	TFN	PA(TF), PSf(S)	RO	BSA	∼70${}^{{}^{\circ}}$	11.1% flux decrease, resistance against chlorination	[30]
Ti_3_C_2_T_*x*_	Stacked	AgNo_3_, PVDF(S)	NF	BSA, MB	35${}^{{}^{\circ}}$	FRR = 97%	[204]

(C) cross-linker; (S) substrate; (TF) thin film; LbL layer-by-layer; FRR flux recovery ratio; *R_ir_* irreversible fouling.

**Table 2:** Photocatalytic nanosheet membranes to mitigate non-migratory fouling.

Nanosheet	Type	Materials	Application	Organic Dye	Light	WCA	Highlights	Ref.
GO(CC)	Stacked	TiO_2_-NT(P), Ag-NP(CC), cellulose(S)	-	MB	Vis	-	complete flux decline, twice the flux of membrane without irradiation	[205]
GO(CC)	Stacked	TiO_2_-NP(P), MCE(S)	UF	DB, MO	UV, Vis	11${}^{{}^{\circ}}$	no irreversible fouling	[31]
N-GO(CC)	MMM	TiO_2_-NP(P), PSf(S)	UF	MB	UV, Vis	59.2 ± 1.2${}^{{}^{\circ}}$	FRR-UV = 94.6%, FRR-vis = 90.1%	[206]
rGO(CC), TiO_2_(P)	Stacked	Al_2_O_3_(S)	NF	MB, RhB, Congo Red, MO	Vis	29.3 ± 3.4${}^{{}^{\circ}}$	nearly constant permeance and selectivity	[207]
LDH(CC), gCN(H)(CC)	MMM	Ag_3_PO_4_(P), NH_2_−Ag_3_PO_4_(P), PES(S)	MF-MBR	BSA, AO7	Vis	40${}^{{}^{\circ}}$–50${}^{{}^{\circ}}$	highest removal under light irradiation was for LDH-Ag	[208]
BP	MMM	PSf(S), SPEEK(S)	NF	MB	UV, Vis	increase	FRR = 85%	[209]

(CC) co-catalyst; (P) photocatalyst; (S) substrate; FRR flux recovery ratio.

**Figure 8:**
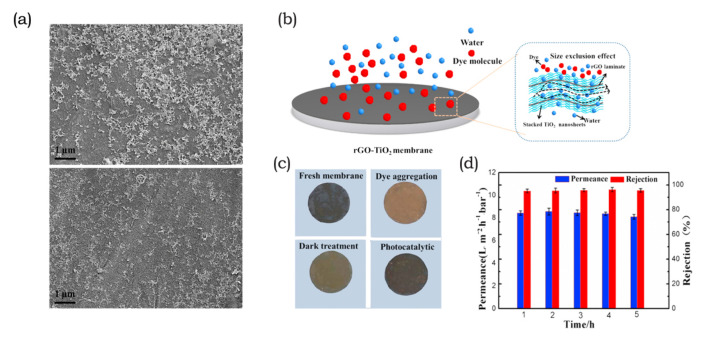
(**a**) Fouled membrane surfaces after testing: unmodified PPA membrane fouled by BSA (top); and, improved BSA fouling resistance of BN-modified PPA (bottom). Adapted from [203], Copyright (2019), with permission from Elsevier. (**b**–**d**) Illustration of a flat sheet nanosheet membrane separating dye molecules based on the sieving effect. The coloration of the flat sheet stacked rGO/TiO_2_ membrane, before testing, after dye aggregation, after washing under dark conditions, and almost complete recovery after photocatalytic cleaning. Rejection was maintained with operation cycles whereas permeance slightly decreased. Adapted from [207], Copyright (2020), with permission from Elsevier.

Boron nitride (BN) is another material that is receiving an increasing amount of attention in the field of water purification membranes. BN was tested by Abdikheibari et al. [203] to improve the antifouling performance of stacked TFC membranes. Amine-functionalized BN nanosheet membranes showed a negligible increase in membrane thickness of only a few nm. As a result, the surface bacme smoother with a roughness of 6.13 ± 0.56 nm. The increased hydrophilicity of the modified membrane resulted in a pure water flux increase of 59%. When compared with the 24.2% fouling rate of the pristine membrane, A-BN modification reduced the fouling rate to 11.9% plus a small amount of irreversible fouling. Figure 8a displays the improved fouling resistance of PPA and PPA-BN membranes fouled with BSA. A diminished propensity for fouling was identified for the modified membranes. Final experiments confirmed the strong adhesion of nanosheets to the membrane surface, and showed that no nanosheets were detached [203]. By contrast in the previous study of Abdikheibari et al. [210], aminated BN nanosheets were embedded in a PPA thin layer, and achieved flux improvement and enhanced fouling resistance of 13.4% and 5.2%, respectively. These results highlight the importance of surface exposure of nanosheets for stacked TFC membranes [203]. In their most recent work, Abdikheibari et al. [211] achieved even higher flux improvement of 69% by simultaneously embedding and coating aminated-BN nanosheets in and on a PPA thin film layer. In addition to the improved flux, the enhanced NOM rejection resulted in a lower chlorine demand for the permeate.

**Figure 9:**
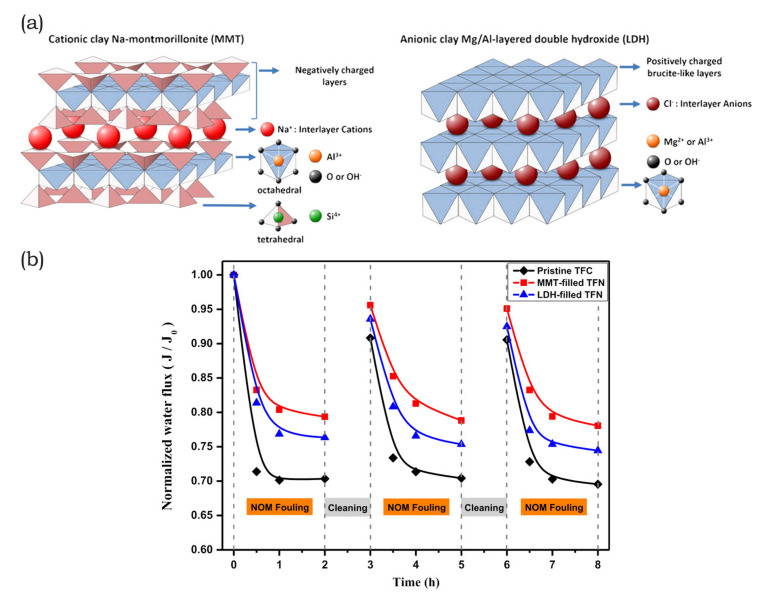
(**a**) Different structures of the cationic clay montmorillonite (MMT), and anionic clay LDH. (**b**)Normalized water flux during membrane testing showing different results based on pristine and modified membranes. Reprinted from [200], Copyright (2015), with permission from Elsevier.

Dong et al. [200] conducted the first comparison of cationic and anionic clay nanosheet fillers to rate the respective suitability of antifouling agents in MMM. In their work, they embedded either Na-montmorillonite (MMT) or Mg/A-LDH in the PA layer of a TFN RO membrane. They investigated the effect that different nanosheet structures exert on separation performance as shown in Figure 9a. Surface roughness increased with nanosheet incorporation as they affected the nodular structures of the PA layer. However, the leaf-like structures in MMT-TFN membranes increased the roughness by comparison with the nodular-like structures of LDH-TFN. Both types of clay nanofillers effectively decreased the water contact angle with an increase in nanosheet concentration. However, MMT increased the negative membrane surface charge, whereas LDH reduced it by comparison with the pristine TFC membrane. The zeta potential was negative in the pH range of four to 10 and increased with the pH due to a deprotonation of the carboxyl groups on the membrane surface. Generally, MMT-TFN exhibited higher flux rates, while both nanosheet fillers experienced a flux decline due to agglomeration at high concentrations. Fouling testing with BSA demonstrated that both of the nanofillers improved the antifouling properties of the membrane. Interestingly, MMT-TFN showed a slower flux decline due to the electrostatic repulsions of a negatively charged membrane surface and foulant. Additionally, the fouling propensity of the membranes tested with the cationic surfactant DTAB showed that the less negatively charged LDH-TFN membrane had a lower flux decline. In the presence of TA as a NOM foulant, MMT-TFN was superior to both LDH-TFN and the pristine membrane due to the negative charge of the foulant. Repeated cleaning and fouling cycles demonstrated the improved antifouling propensity of MMT-TFN membranes by comparison with the pristine and LDH-TFN counterparts as shown in Figure 9b. Dong et al. [200] demonstrated the importance of surface charge in terms of fouling propensity and the choice of nanofillers in accordance with their intended application. Another approach to modify membrane surfaces is the use of zwitterions, as reported in the work of Rahimi and Mahdavi [160]. The substrate structure developed wider pores and larger pore channels because of the presence of zwitterions grafted onto nanosheets. With the observed decrease in surface roughness upon blending with GO-g-PMSA, the wettability increased. During the phase separation, GO-g-PMSA migrated to the membrane surface and exposed the zwitterions to the feed. As a result, BSA foulants were inhibited from aggregating on the membrane surface [160]. Membrane functionalization with zwitterions grafted onto GO has also been reported in other studies [157,159,212]. Besides modification with zwitterions to improve nanosheet migration towards the membrane surface, the application of an electric or magnetic field is another approach to better surface exposure [121,213].

GBN are most often applied as photocatalytic membranes, followed by gCN(H). The limited photocatalytic activity of GO and rGO compels them to act as charge separators to inhibit electron-hole recombination or as anchor sites for photocatalysts to improve their dispersion. For example, TiO_2_ is a popular photocatalyst and is most often employed as a nanoparticle in combination with GO nanosheets, as reported in several publications [31,214,215,216,217]. However, 1D TiO_2_ nanostructures exhibit improved photocatalytic properties due to a larger specific surface area. Thus, an increase in contact area that is more available for adsorption of pollutants or to form heterostructures with other nanomaterials [83,218,219]. For example, Liu et al. [205] tested Ag-NP/GO/TiO_2_-NT nanocomposite coated membranes for photocatalytic properties that could alleviate non-migratory fouling. TiO_2_-NTs formed porous particles encapsulated by GO when forming layered structures. Ag particles were well dispersed on the nanotubes and nanosheets, and the nanostructures were vacuum-filtered onto a flat-sheet cellulose substrate. The formation of heterostructures decreased the bandgap of TiO_2_ and, hence, increased its visible light absorbance. Due to the high adsorption rate for dyes on GO and TiO_2_, the flux decline was higher under dark conditions than under visible light irradiation. In their work, they also compared the membrane performance with that of Ag-NP/GO/P25, which showed lower adsorption photocatalytic properties [205]. In a comparison with 1D TiO_2_ structures, Yu et al. [207] reported the photocatalytic membranes of 2D/2D, rGO/TiO_2_ heterostructures. In their work, micron-sized rGO operated as a template for the immobilization of nanosized TiO_2_ sheets. These were stacked on a flat-sheet Al_2_O_3_ support, as illustrated in Figure 8b. The advantages of this 2D/2D nanostructure included a controlled dispersion of TiO_2_ in order to avoid agglomeration, and an induced charge separation for enhanced photocatalytic activity. After light irradiation, the membranes recovered flux and MB rejection levels near the initial values, hence suggesting long-term stability against dye fouling as indicated in Figure 8c,d. Membrane performance was maintained because of the combination of many adsorption sites on the highly porous nanostructure and the generation of reactive oxygen species [207].

Besides coating with nanosized photocatalysts, self-cleaning conventional MMM and TFN were reported to also employ LDH and BP in contrast to the more frequently reported gCN(H) [220,221,222,223]. The laminar structure of the bulk material of phosphorene is that of black phosphorus, which was immersed in SPEEK membranes. That induced an increase in the water contact angle of from 48.3 ± 0.67${}^{{}^{\circ}}$ to 81.5 ± 0.64${}^{{}^{\circ}}$ due to the rather hydrophobic nature of the nanosheets. The blended membranes also showed pore sizes that were less uniform than of the pristine membrane. However, intermittent UV irradiation enabled flux recoveries of approximately 85% for the modified membrane when compared with only 14% for pure SPEEK [209]. In order to elucidate the performance of individual nanofillers as photocatalysts, Ghalamchi et al. [208] compared ZnAlCu–NLDH/PES, gCN(H)/PES, Ag_3_PO_4_/ZnAlCu–NLDH/PES, and NH_2_–Ag_3_PO_4_/gCN(H)/PES mixed matrix membranes. Blending with LDH resulted in a more porous structure, whereas gCN(H) remained mostly within the matrix and thus formed a denser membrane with lower porosity. Surface roughness was higher with a lower pore size distribution in LDH membranes. Permeability testing indicated that due to the increased migration of LDH to the surface, pore blocking occurred, and the water flux was decreased. However, the addition of nanofillers improved flux recovery compared with that of the pristine membrane. These results can be explained with the overall increase in the hydrophilicity and the formation of a hydration layer, which hampered the adhesion of BSA. Further experiments that analyzed the leaching of Ag and Zn, demonstrated how the presence of gCN(H) stabilized Ag. In addition, high rates of Zn release were observed, which can be explained by the increased migration towards the surface during membrane fabrication. The results from the photocatalytic degradation of dye pollutants indicate a synergistic effect of the heterostructures. As a result, heterjunctions render Ag_3_PO_4_ and NH_2_Ag_3_PO_4_, as stronger photocatalysts due to the presence of the nanosheets.

### 4.2. Spreadable Fouling Strategies

The majority of nanosheets employed to mitigate oil fouling have been either GO or rGO, as shown in Table 3. In some cases, gCN(H) and Bi_12_O_17_Cl_2_, LDH, MXene, and CuO were employed. Additionally, the majority of the reported literature describes either stacked or grafted membranes rather than MMM. For the blended nanocomposite membranes, nanosheets were, in most cases, embedded in a PDA layer on the membrane surface. Two approaches were found in the literature to alleviate oil fouling. One used the design of superhydrophilic and superoleophobic membrane surfaces. The other involved creating photocatalytic membranes that would destroy pollutants and increase hydrophilicity. Nanosheets were employed as photocatalysts [224], heterostructures [224], binding sites for nanoparticles [225], or modifiers for surface properties, such as hydrophilicity and roughness.

**Figure 10:**
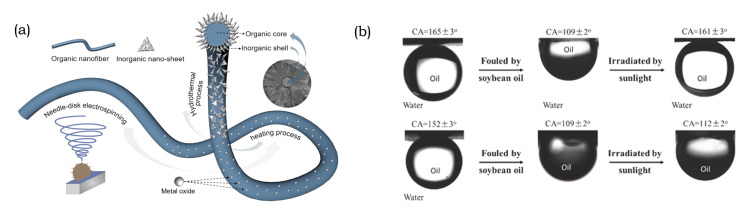
(**a**) Fabrication of an electrospun core-shell nanofiber. Reproduced with permission from [228], published by MDPI AG. License CC-BY. (**b**) Changes of the oil contact angle from before membrane testing, to after testing and after photocatalytic cleaning. From left to right, the changing OCA for GO/gCN(H)@TiO_2_ and GO. Copyright (2018) Wiley. Adapted and used with permission from [155].

The use of nanosheets as antifouling membranes has advantages that extend beyond their photocatalytic activity [229]. There are alternative membrane designs. One alternative involves the in-situ growth of CuO nanosheets on the surface of eletrospun polymers [228], as shown in Figure 10a. Another alternative uses nanosheet coatings with polymer brushes grafted onto LDH [111]. The former exhibits a water contact angle of 0${}^{{}^{\circ}}$ that is attributed to the high surface roughness and hydrophilicity of CuO nanosheets. That structure traps water between the nanosheets and, thus, forms a barrier for oil. Rejection of several types of oils was above 99.8% with a flux of 250 L m^−2^h^−1^, which was retained for 80 min and indicated the good fouling properties of the membrane [228]. Another type of anti-oil-fouling membrane was fabricated by stacking SiO_2_-decorated rGO nanosheets onto a support, and coating it with a layer of hydrophilic PDA. The nanoparticles created nanochannels that increased the permeability, while the surface charge retained the rejection rate. Additionally, the water contact angle was near 0 ${}^{{}^{\circ}}$ and, thus, formed a repulsive water layer to impede oil adhesion [225].

For MMM, Qin et al. [130] reported a MMM that was resistant to spreadable foulants by blending GO nanosheets into a polymer. That same polymer was subsequently coated with a hydrogel to reduce the surface roughness. The presence of GO during phase inversion resulted in higher porosity in the support structure and in an increased pore density in the selective layer. Hydrogel was employed to fill the valleys caused by faster phase inversion. As a result, the membrane hydrophilic increased with a water contact angle of 30.5 ± 3.3${}^{{}^{\circ}}$ as well as an underwater oil contact angle of 141.6 ± 3.5${}^{{}^{\circ}}$. In terms of the rejection of oil-surfactants and fouling in response to an increase in pressure, the hydrogel-functionalized GO membrane demonstrated outstanding performance [130].

The antifouling photocatalytic membranes employed heterostructures with gCN(H) as a photocatalyst and GO as a co-catalyst for hampering electron-hole pair recombination [224]. Other groups have reported the immobilization of TiO_2_-NP as a photocatalyst on gCN(H) nanosheets that were further intercalated between layers of GO to improve stability [155]. Heterostructures between Bi_12_O_17_Cl_2_−TiO_2_NW and rGO coated by PDA also enhanced the photocatalytic performance of the membranes [219]. Nishimoto et al. [231] demonstrated the self-cleaning effect of photocatalytic membranes in terms of spreadable foulants. They reported that TiO_2_-coated surfaces showed significant self-cleaning properties when irradiated with UV light under a continuous water flow. Yu et al. [219] agreed with these results after developing photocatalytic membranes of rGO/PDA/Bi_12_O_17_Cl_2_−TiO_2_NW sheet-like nanohybrids that were vacuum filtered on a CA support. These membranes demonstrated constant flux and efficient separation of dye-oil mixtures under visible light irradiation. The membrane surface intercepted oil particles from the feed solution, thus improving oil separation, and showed self-cleaning properties upon light irradiation. By contrast, the same membrane operated in dark exhibited a steady decline in pollutant rejection and flux. Upon light irradiation, eletrons transfer from Bi_12_O_17_Cl_2_ to TiO_2_ and further to rGO, where they form superoxide free radicals in the presence of free oxygen molecules. Holes formed on the catalyst produce hydroxyl radicals with water. In addition, rGO adsorbs dye molecules that are degraded by the generated radicals. Therefore, the good photocatalytic performance of the RGO/PDA/Bi_12_O_17_Cl_2_−TiO_2_−CA membrane is attributed to the fast electron transfer and molecule adsorption [219].

**Figure 11:**
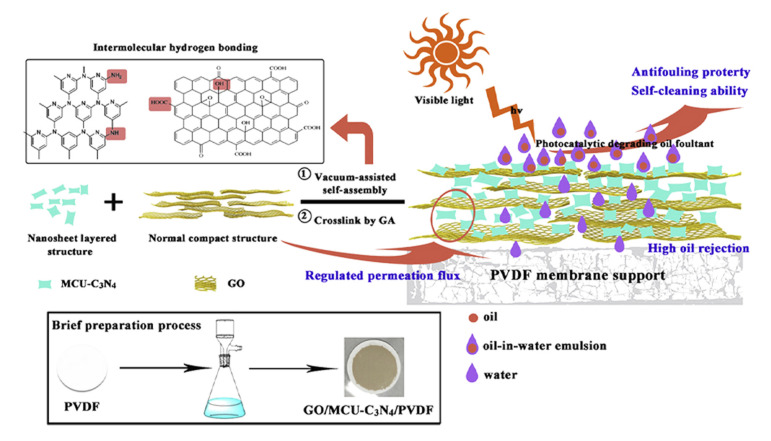
The fabrication process of photocatalytic gCN(H) membranes. Reprinted from [224], Copyright (2019), with permission from Elsevier.

The antifouling and membrane separation performances of nanocomposite membranes were compared in terms of TiO_2_ nanowire or nanoparticle intercalation of GO nanosheets. Due to the increase in surface area, the adsorption and resultant photocatalytic removal of pollutants was better for NW than for NP. Additionally, the hydrophilicity and flux were enhanced with intercalated 1D nanostructures [230]. Shi et al. [224] reported that self-cleaning membranes with photocatalytic properties reduce irreversible fouling. The membrane fabrication process via vacuum filtration, which includes the resultant laminar structure with MCU-gCN(H)-intercalated GO, is illustrated in Figure 11. The MCU-gCN(H) membranes suffered from severe fouling and exhibited limited flux recovery after physical cleaning. However, the performance was significantly improved after visible light irradiation. Irreversible fouling led to a continuous decrease in flux after every cycle for different types of oil-surfactant foulants. Nonetheless, intermittent light irradiation increased the FRR for all feed solutions to above 88%. The superoxide radical and hydroxyl radicals, generated on the GO and MCU-gCN(H) surfaces, respectively, degraded the dye molecules and thus improved the membrane performance. Liu et al. [155] developed another example of photocatalytic anti-oil fouling membranes, and involved a free-standing GO membrane intercalated with gCN(H)@TiO_2_-NP. In contrast to the prepared membrane of Shi et al. [224], TiO_2_-NP were added to increase the interlayer distance between the stacked nanosheets, rendering the membrane surface rough and improving hydrophilicity. As a result, water was trapped within the structure and increased the underwater oil contact angle to 170${}^{{}^{\circ}}$, which inhibited the adhesion of oil droplets. However, an excess of nanoparticle loading blocked the water passage within the interlayer channels and increased the membrane thickness, resulting in a decrease in water flux. The self-cleaning ability was tested with visible light irradiation after testing the separation of oil/surfactant-water emulsions. Spreadable foulants were not effectively removed with physical cleaning, but photo-assisted washing achieved almost complete flux recovery. In order to analyze the effect of the gCN(H)@TiO_2_ photocatalyst, oil contact angle measurements were conducted for GO and GO/gCN(H)@TiO_2_ membranes. Figure 10b demonstrates high initial OCA the nanohybrid membranes (top figures). Applying intermittent light irradiation almost completely recovered the initial OCA after fouling with soybean oil. In contrast, GO membranes (bottom figure) initially had a lower but similar OCA after fouling, which could not be recovered by photocatalytic cleaning.

### 4.3. Inorganic Fouling Strategies

Fewer studies have focused on the modification of membranes for scaling mitigation when compared with organic or biofouling. Nonetheless, the available literature indicates that a combination of membrane integration and conventional scaling strategies can effectively alleviate scaling. The literature references to 2D nanocomposite membranes are very limited and mostly focuses on GO nanosheet membranes.

Ray et al. [232] prepared GO nanosheet membranes to simultaneously address bio, organic, and inorganic fouling. However, in their work, GO nanosheets improved the antifouling properties of membranes as nucleation sites for other nanomaterials. They immobilized GO on a PA membrane used in RO via esterification. Subsequently Au nanostars were formed on the nanosheets, using an in-situ growth method. In their study the PA-GO modified membrane exhibited higher scaling due to a negative surface charge that attracted Ca^2+^ ions [232].

**Figure 12:**
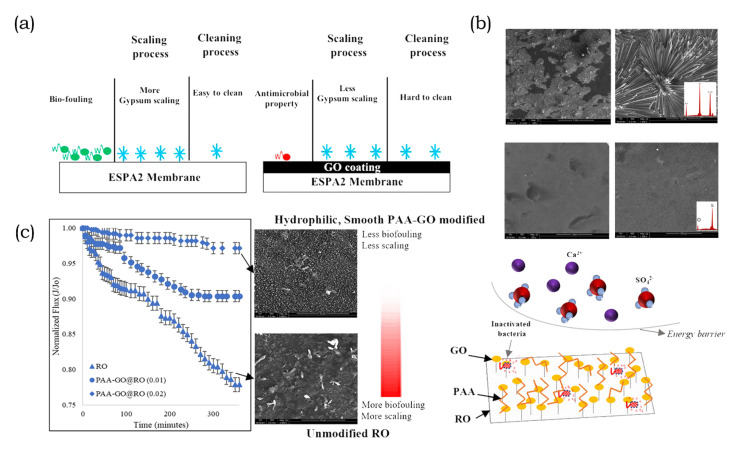
(**a**) I mproved biofouling and scaling propensity, but reduced flux recovery of GO@PA membranes. Reprinted from [233], Copyright (2018) with permission from Elsevier. (**b**,**c**) Scale layer on the PA RO membrane and the characteristic needle-like crystals shown in the top two pictures. The bottom pictures demonstrate the successful antiscaling PAA-GO functionalized membrane: normalized flux, membrane surface, and illustrated membrane surface modification. Adapted from [234], Copyright (2020) with permission from Elsevier. License CC-BY.

In contrast to the work of Ray et al. [232], Cao et al. [233] described the fabrication of a GO-functionalized PA RO membrane with improved anti-scaling properties. With membrane modification, the root mean surface roughness increased from 77.1 ± 4.8 to 113.9 ± 8.5 nm. However, the water contact angle and surface zeta potential decreased from 82.2 ± 1.3${}^{{}^{\circ}}$ to 43.1 ± 4.2${}^{{}^{\circ}}$, and from −20.8 ± 0.4 mV at pH = 5.80 to −38.0 ± 0.2 mV, respectively. The negative surface charge enabled the repulsion of negatively charged gypsum particles. Simultaneously, the hydrophilicity increased the energy barrier for precipitation on the surface. Despite the improved fouling properties with the addition of GO, these membranes showed a lower removal rate of gypsum after cleaning with water. Thus, the flux recovery of the pristine membrane was about 78.6 ± 6.6% and 69.6 ± 5.4% for the GO membrane under saturated conditions. This effect was the result of the higher density of carboxyl groups on the GO nanosheet, which bonded with Ca^2+^ ions. The effect of the membrane modified by this group is illustrated in Figure 12a. [233].

Ashfaq et al. [234] reported the fabrication of GO-coated polyamide RO membranes similar to those from Cao et al. [233]. In contrast to their previous work [235], they immobilized polymerized acrylic acid in addition to GO on the RO membrane. When compared to the RO modification with GO only, the antiscalant further decreased the roughness to 61.555 nm. Upon functionalization, the water contact angle was further decreased to 24.4 ± 1.3${}^{{}^{\circ}}$ and, thus, enhanced the wettability as well as the hydrophilicity. The modified membranes demonstrated a steady-state flux development after 250 min. Both the rate of attachment and the detachment of foulants were in equilibrium. The decline in flux was 3% for the higher and concentration of PAA and 10% for the lower. Because of membrane functionalization, scaling was inhibited and no crystals were formed on the surface as shown in the bottom two pictures in Figure 12b. In contrast, the upper two pictures show the scale formation on pristine membranes [234]. Figure 12c, shows that the addition and further increase in PAA-GO resulted in less biofouling and scaling while improving the normalized flux. Improved fouling resistance was achieved, as validated by SEM, although the surface of the structure only shows a partial covering of the membrane surface with PAA and GO.

## 5. Challenges

As established in previous sections, nanosheets are promising building blocks for membranes to render them resistant to fouling. However, nanosheet membranes, especially fouling resistant membranes, are a comparably new research field. Therefore, several challenges remain unsolved. Generally, surface-exposure of nanosheets is key to obtain antifouling membranes. Nevertheless, large-area stacked nanosheet membranes were seldom reported. Thus far, the majority of publications focus on lab-scale experiments. The large-scale production of TFN and conventional MMM would be easier due to incorporation of exisiting fabrication lines. However, stacked TFC and stacked nanosheet membranes are more difficult to up-scale. Additionally, most reports feature flat-sheet membranes, whereas tubular or hollow-fibre membranes also find wide application in the real world. The synthesis of nanosheet membranes is also challenged by the complex and multiple fabrication steps. In many reports, the 2D nanomaterials required further functionalization. These additional modifications are necessary to improve their stability in solution or on the membrane. Moreover, nanosheet membranes often have complex designs involving several nanomaterials. It is recognized that 2D nanomaterials can effectively address biofouling due to their bactericidal properties. Nonetheless, the effect of such materials on the human body might be different and it is subject to ongoing studies. Before nanosheet membranes are used to treat water for human consumption, their toxicity must be fully elucidated. Additionally, long-term testing is paramount in determining the membrane and nanosheet stability.

## 6. Summary and Future Perspectives

In this review, the development of nanosheet membranes is summarized based on the classification of conventional MMM, stacked, stacked TFC, and TFN membranes. It is apparent that 2D nanosheets play an important role in promoting antifouling during water purification by improving hydrophilicity, surface roughness, zeta potential, and photocatalytic destruction of foulants via photocatalysis. The present work demonstrated improved fouling alleviation for non-migratory, spreadable, and inorganic foulants due to the presence of nanosheets. Although their bactericidal properties have been widely investigated, biofouling was not covered in this work.

Systematic research via the "Web of Science" indicated an ongoing upward trend for nanosheet membranes and recent interest in their propensity for promoting antifouling. Research activity has increased over the years and it shows the growing awareness of scientists for the promising properties of nanosheets. A combination of this upward trend of nanosheet-related research output and the versatile applications of these materials suggest rapid developments and further discoveries. Interest in these nanostructures is fueled by continuous discoveries of new materials and modifications of synthesis routes. Nonetheless, it is expected that GBN-modified membranes will continue to dominate the field. Additionally, new materials will receive more attention, because GBN materials suffer from shortcomings. Recent developments show that nanohybrids clearly outperform single nanomaterials and tend to dominate current research efforts. However, thus far, most nanosheet-related reviews feature nanoparticles or graphene-based materials, as they dominate the research output in this field.

As the present work shows, many nanosheet materials are suitable for use in the improvement of membrane performance and for imparting antifouling activities. In particular, TMDs, MXene, and BN are promising alternatives in terms of permselectivity. Clearly, most researchers focus on passive antifouling strategies by employing hydrophilic materials, whereas photocatalytic membranes are less studied. Photocatalytic properties of certain nanomaterials are well documented and they are being intensively studied by many groups. However, integrated separation and photocatalytic testing of membranes for water purification have been limited. The photocatalytic activity of 2D nanomaterials and separation using nanosheet membranes are tested separately. The intrinsic properties of 2D nanomaterials, make them advantageous for application to integrated membrane operations. The 2D nanomaterials that show promise for the fabrication of photocatalytic membranes for water treatment include gCN(H), metal oxides, and GBN. In addition to increasing separation and permeation, 2D nanomaterials also improve rejection, membrane longevity, washing intervals, and the destruction of CEC. As established in other publications, there is an infinite number of combinations of nanomaterials for photocatalytic nanohybrid heterostructures. That gives membrane researchers many possibilities for the development of photocatalytic membranes. Despite promising results, more work is required in order to address difficulties in operations and in up-scaling production. Another finding indicates that most studies have focused on the mitigation of organic fouling, particularly non-migratory fouling. In contrast, spreadable fouling has received less investigation as it requires the formation of amphiphilic surfaces, which are achieved mostly via photocatalytic testing with nanosheets. However, the alleviation of inorganic fouling by employing 2D nanomaterials has been documented by few studies, but has been limited to GO. These studies show that nanomaterials can effectively create an energy barrier that hampers inorganic fouling. Additionally, they indicate the suitability of nanosheets to overcome the drawbacks of GO-modified membranes. Overall, nanosheet membranes have versatile structures and properties as a result of the different types of nanomaterials employed.

The improvement of conventional membranes by employing 2D nanomaterials effectively tackles the permeability-selectivity bottleneck while providing mechanical stability and alleviating fouling. Different types of nanosheets are recommended for different applications. It is preferable to stack photocatalytic membranes in order to maximize surface exposure. In addition, high levels of nanosheet exposure are also recommended to produce membranes that are resistant to spreadable foulants. During RO and FO, stacked TFC and TFN show promising results in resisting damage by chlorine and alleviating scaling. Conventional MMM are suitable for MF and UF when no top layer is present, and for passive strategies in general. Stacked nanosheet membranes are suggested for UF and NF, for photocatalytic applications in general, and for biofouling mitigation.

Taking all of these new developments, discoveries, and improvements into consideration, an infinite amount of different nanosheet membranes is possible. As can be seen, the field of nanosheet membranes is highly innovative and subject to rapid development. That makes it difficult for scientists to keep track of all the relevant breakthroughs. Taking the recent developments into consideration, it is to believe that nanosheet membranes, especially antifouling membranes, are merely in their infancy. In the future, the key for efficient antifouling nanosheet membranes is interdisciplinary research. This will yield outstanding membranes with a wide range of applications.

## Figures and Tables

**Table 3:** Nanosheet Membranes to mitigate spreadable fouling.

Nanosheet	Type	Materials	Foulant	WCA	Highlights	Ref.
**Normal Testing**						
GO	Stacked	PDA(C), halloysite-NT, PEN(S)	n-hexane-in-water emulsion	0${}^{{}^{\circ}}$	OCA = 136 ± 2${}^{{}^{\circ}}$, stable at high temperatures, electrospun support	[226]
GO	MMM	PVA, PES(S)	surfactant/sunflower oil and olive oil mixture	30.5 ± 3.3${}^{{}^{\circ}}$	OCA = 141.6 ± 3.5${}^{{}^{\circ}}$	[130]
rGO	Stacked	PDA(C), MCE(S)	1,2-dichlorethane, toluene, n-hexane, diesel	near 0 ${}^{{}^{\circ}}$	OCA = 156.1±1.2${}^{{}^{\circ}}$	[227]
rGO	Stacked	PDA(C), SiO_2_-NP, PVDF(S)	diesel oil/water emulsion	0${}^{{}^{\circ}}$	OCA = 130${}^{{}^{\circ}}$, FRR = 87.2%	[225]
CuO	MMM	PVDF-HFP(S)	olive oil, cooking oil, lubricant oil	0${}^{{}^{\circ}}$	OCA = 152.4${}^{{}^{\circ}}$, electronspun polymer with nanosheet shell	[228]
Ti_3_C_2_T_*x*_	Stacked	white print paper(S)	sunflower oil, diesel oil, silicon oil, petroleum ether, hexane	0${}^{{}^{\circ}}$	OCA = 137${}^{{}^{\circ}}$	[229]
**Photo-Assisted Testing**						
GO(CC)	Stacked	PDA(C), TiO_2_-NW(P), CA(S)	MB and diesel oil/gasoline/ dichloro–methane–water emulsion	48.1 ${}^{{}^{\circ}}$	visible light, OCA = 132${}^{{}^{\circ}}$	[230]
rGO(CC), Bi_12_O_17_Cl_2_(P)	Stacked	PDA(C), TiO_2_-NW(CC), CA(S)	MB and diesel oil/water emulsion	55.74 ${}^{{}^{\circ}}$	visible light	[219]
GO(CC), gCN(H)(CC)	Stacked	TiO_2_-NP(P)	soy-bean oil	43${}^{{}^{\circ}}$	visible light, OCA = 170${}^{{}^{\circ}}$, FRR = 95%	[155]
GO(C), MCU-CN(H)(P)	Stacked	GA(C), PVDF(S)	SDS-diesel oil /petroleum-ether/ dichloromethane/hexane in water	48.87 ${}^{{}^{\circ}}$	visible light	[224]

(C) cross-linker; (CC) co-catalyst; (P) photocatalyst; (S) substrate; (TF) thin film; FRR flux recovery ratio.

## Abbreviations

The following abbreviations are used in this manuscript:

(C)	Cross-linker
(CC)	Co-Catalyst
(P)	Photocatalyst
(S)	Substrate
(TF)	Thin Film
AAO	Anodic aluminium oxide
AO7	Acid orange 7
AOP	Advanced oxidation processes
BN	Boron nitride
BP	Black phosphorus
BSA	Bovine Serum Albumin
CA	Cellulose Acetate
CB	Conduction band
CEC	Contaminants of concern
CNT	Carbon nanotube
COF	Covalent organic framework
DB	Diphenhydramine
DBP	Disinfection-by-products
DTAB	Dodecyltrimethylammonium bromide
FO	Forward osmosis
FRR	Flux recovery ratio
GA	Glutaraldehyde
GBN	Graphene-based-nanomaterials
gCN(H)	Graphitic carbon nitride
GO	Graphene oxide
HA	Humic acid
IP	Interfacial polymerization
LbL	Layer-by-Layer
LDH	Layered double hydroxide
MB	Methylene blue
MBR	Membrane Batch Reactor
MCE	Methyl Cellulose Ester
MF	Microfiltration
MMM	Mixed matrix membrane
MMT	Montmorillonite
MO	Methyl orange
MOF	Metal organic framework
MPD	M-phenylenediamine
MWCO	molecular weight cut-off
NF	Nanofiltration
NHE	Normal hydrogen electrode
NIPS	Non-solvent induced phase separation
NOM	Natural organic matter
NP	Nanoparticle
NT	Nanotube
NW	Nanowire
OCA	Oil contact angle
PA	Polyamide
PAA	Polyacrylic acid
PAI	Polyamide-imide
PAN	Polyacrylonitrile
PDA	Polydopamine
PDDA	Poly(diallyldimethylammonium chloride)
PEI	Polyethylenimine
PEN	Poly(arylene ether nitrile)
PES	Polyethersulfone
PMR	Photocatalytic membrane reactor
PMSA	Poly 2-(methacryloyloxy)ethyl dimethyl-(3-sulfo-propyl)ammonium hydroxide
PPA	Polypiperazine amide
PSf	Polysulfone
PVA	Polyvinyl alcohol
PVDF	Polyvinylidene difluoride
PVDF-HFP	Poly(vinylidene fluoride-co-hexafluoropropylene)
rGO	Reduced graphene oxide
RhB	Rhodamine B
RO	Reverse osmosis
ROS	Reactive oxygen species
SA	Sodium alginate
SDG	Sustainable Development Goal
SDS	Sodium dodecyl sulfate
SPEEK	Sulfonated poly(ether ether ketone)
TA	Tannic acid
TEOA	Triethanolamine
TFC	Thin film composite
TFN	Thin film nanocomposite
TMC	trimesoyl chloride
TMD	Transition metal dichalcegonides
TMO	Tranisition metal oxides
UF	Ultrafiltration
VB	Valence band
WCA	Water contact angle
